# Revealing formate production from carbon monoxide in wild type and mutants of Rnf‐ and Ech‐containing acetogens, *Acetobacterium woodii* and *Thermoanaerobacter kivui*

**DOI:** 10.1111/1751-7915.13663

**Published:** 2020-09-21

**Authors:** Fabian M. Schwarz, Sarah Ciurus, Surbhi Jain, Christoph Baum, Anja Wiechmann, Mirko Basen, Volker Müller

**Affiliations:** ^1^ Molecular Microbiology and Bioenergetics Institute of Molecular Biosciences Johann Wolfgang Goethe University Frankfurt am Main Germany; ^2^ Microbiology Institute of Biological Sciences University Rostock Rostock Germany

## Abstract

Acetogenic bacteria have gained much attraction in recent years as they can produce different biofuels and biochemicals from H_2_ plus CO_2_ or even CO alone, therefore opening a promising alternative route for the production of biofuels from renewable sources compared to existing sugar‐based routes. However, CO metabolism still raises questions concerning the biochemistry and bioenergetics in many acetogens. In this study, we focused on the two acetogenic bacteria *Acetobacterium woodii* and *Thermoanaerobacter kivui* which, so far, are the only identified acetogens harbouring a H_2_‐dependent CO_2_ reductase and furthermore belong to different classes of ‘Rnf’‐ and ‘Ech‐acetogens’. Both strains catalysed the conversion of CO into the bulk chemical acetate and formate. Formate production was stimulated by uncoupling the energy metabolism from the Wood–Ljungdahl pathway, and specific rates of 1.44 and 1.34 mmol g^−1^ h^−1^ for *A. woodii ∆rnf* and *T. kivui* wild type were reached. The demonstrated CO‐based formate production rates are, to the best of our knowledge, among the highest rates ever reported. Using mutants of *∆hdcr*, *∆cooS*, *∆hydBA*, *∆rnf* and *∆ech2* with deficiencies in key enzyme activities of the central metabolism enabled us to postulate two different CO utilization pathways in these two model organisms.

## Graphical abstract

In this study we focused on the two acetogenic bacteria *Acetobacterium woodii* and *Thermoanaerobacter kivui* and demonstrated their potential as whole cell biocatalysts for the conversion of CO into the bulk chemicals acetate and formate. Notably, no external electron donor such as H_2_ was required for the conversion of CO. We could further shed light into the dark of CO utilization in *A. woodii* and *T. kivui* by using mutants with deficiencies in key enzyme activities of the central metabolism.

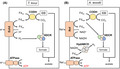

## Introduction

Most bulk chemicals are still based on fossil fuels, such as crude oil and natural gas. But in times of global climate change and the fear of dwindling resources, the development of sustainable biological methods for the production of industrially relevant chemicals is urgently needed. Historically, biotechnological production plants for chemicals were based on sugar (i.e. sugarcane, corn and wheat) (Naik *et al*., 2010) and there is a broad range of processes for the production of different compounds from different sources by different organisms (Wendisch, 2014). However, the processes have in common that they also produce CO_2_ and that they compete with the food industry for the same feedstock (Fargione *et al*., 2008; Ajanovic, 2011). Second‐generation biofuels are based on lignocellulose (Naik *et al*., 2010; Kucharska *et al*., 2018), but, again, the process is not CO_2_ neutral and due to technical and non‐technical barriers most projects of this kind were put on hold (Padella *et al*., 2019). Third‐generation biofuels are based on carbon oxides as feedstock and instead of producing CO_2_ gas fermentation captures and stores CO_2_ in the form of value‐added chemicals (Munasinghe and Khanal, 2010; Dürre and Eikmanns, 2015; Liew *et al*., 2016b; Bengelsdorf and Dürre, 2017). One available CO_2_ source is synthesis gas (syngas) which mainly consists of H_2_, CO_2_ and CO. Syngas can be obtained from industrial exhaust gases, such as steel mill off‐gas (Köpke *et al*., 2011) or by gasification of biomass and waste streams, such as sewage sludge and municipal waste (Hammerschmidt *et al*., 2011; Rokni, 2015).

Many microbes are known to convert syngas into chemicals (Henstra *et al*., 2007b; Bengelsdorf *et al*., 2013; Bengelsdorf *et al*., 2018). Among those are the acetogenic bacteria that grow autotrophically by converting H_2_ + CO_2_ to acetate according to Eq. 1:

(1) $$4H_{2}+2{\text{CO}}_{2}↔{\text{CH}}_{3}{\text{COO}}^{‐}+H^{+}+2H_{2}O\;\Delta G_{0}^{\prime }=-95\;\text{kJ}/\text{mol}$$

Some are also able to produce ethanol in addition to acetate (Najafpour and Younesi, 2006; Maddipati *et al*., 2011; Bertsch and Müller, 2015a). Many acetogens can also grow on carbon monoxide according to Eq. 2:

(2) $$4\text{CO}+2H_{2}O↔{\text{CH}}_{3}{\text{COO}}^{‐}+H^{+}+2{\text{CO}}_{2}\;\Delta G_{0}^{\prime }=-165.6\;\text{kJ}/\text{mol}$$

CO is first oxidized to CO_2_ which is subsequently reduced to acetate with electrons derived from CO oxidation (Diekert and Thauer, 1978; Diender *et al*., 2015). The production of acetate and ethanol from syngas requires a linear pathway of CO_2_ reduction that has two branches, the Wood–Ljungdahl pathway (WLP; Drake, 1994; Ragsdale, 2008; Schuchmann and Müller, 2016). In the methyl branch, one molecule of CO_2_ is first reduced to formate, then bound at the expense of ATP hydrolysis to the cofactor tetrahydrofolic acid (THF) and reduced in a THF‐bound form to a methyl group. In the second branch, the carbonyl branch, a second molecule of carbon dioxide, is reduced by the enzyme CO dehydrogenase/acetyl‐CoA synthase (CODH/ACS) to enzyme‐bound CO which is then combined with the methyl group and coenzyme A on the CODH/ACS to acetyl‐CoA. Acetyl‐CoA is then converted *via* acetyl phosphate to acetate generating one ATP (Diekert and Wohlfarth, 1994). Although the ATP yield by substrate level phosphorylation is zero, the bacteria grow by this conversion, due to additional ATP generation by a chemiosmotic mechanism that involves an energized membrane for ATP synthesis (Müller, 2003; Poehlein *et al*., 2012; Schuchmann and Müller, 2014). The respiratory enzymes present in acetogens are either the Rnf or the Ech complex (Biegel and Müller, 2010; Schölmerich and Müller, 2019). Therefore, acetogens can bioenergetically be classified in ‘Rnf‐’ and ‘Ech‐acetogens’ (Schuchmann and Müller, 2014). The translocated ions by these complexes (and the ATP synthase) could either be Na^+^ or H^+^. In *A. woodii,* the Rnf complex translocates Na^+^ and the Ech complex of *T. kivui* translocates H^+^, whereas the Rnf complex of *Clostridium ljungdahlii* most likely pumps H^+^ (Tremblay *et al*., 2012). However, acetogens grow at the thermodynamic limit of life and only a fraction of an ATP is made per turnover (Müller, 2015; Spahn *et al*., 2015; Müller and Hess, 2017).

Acetyl‐CoA (or acetate) can be converted to ethanol, and some acetogens like *Clostridium autoethanogenum* are used industrially to produce ethanol from syngas (Bengelsdorf *et al*., 2013; Takors *et al*., 2018; Köpke and Simpson, 2020). Some acetogens naturally produce minor amounts of butyrate or lactate (Liou *et al*., 2005; Köpke *et al*., 2011; Jeong *et al*., 2015). However, with the advent of genetic tools in acetogens metabolic engineering is now possible leading to many new products like acetone, butanol, 3‐hydroxybutyrate and isopropanol (Köpke *et al*., 2010; Banerjee *et al*., 2014; Bengelsdorf *et al*., 2016; Bengelsdorf and Dürre, 2017). Since acetogens are energy‐limited during growth on H_2_ + CO_2_, minor amounts of the compound of interest are produced along with major amounts of acetic acid. With CO as electron source, the energetics are much better and the selectivity is increased (Bertsch and Müller, 2015a).

Formic acid is produced by many acetogens transiently during acetogenesis from H_2_ + CO_2_ (Peters *et al*., 1999). Formate is an interesting product since it can be further converted by acetogens or other formatotrophic organisms into higher value‐added chemicals (Harris *et al*., 2007; Cotton *et al*., 2019; Hwang *et al*., 2020). Recently, we discovered a novel class of formate dehydrogenases in the acetogens *Acetobacterium woodii* and *Thermoanaerobacter kivui*, namely a hydrogen‐dependent CO_2_ reductase (HDCR) (Schuchmann and Müller, 2013; Schwarz *et al*., 2018). In contrast to classical formate dehydrogenases, HDCR directly uses H_2_ as reductant for CO_2_ reduction for formate. HDCR has a formate dehydrogenase module and a hydrogenase module that are most likely connected by two small FeS centre‐containing electron transfer proteins. HDCR from *A. woodii* and *T. kivui* has extraordinary high rates of CO_2_ hydrogenation, and they are 30‐ and 1200‐times faster than any chemical catalyst (Müller, 2019). Industrially produced hydrogen often contains traces of CO that are tolerated by the enzyme. Indeed, the HDCR purified from *A. woodii* was shown to convert CO in the presence of purified CO dehydrogenase and purified ferredoxin (Schuchmann and Müller, 2013), which may be of physiological and industrial significance. Since the HDCR is oxygen sensitive, a whole cell system for both species was established to capture and store CO_2_ and hydrogen in the form of formic acid (Schuchmann and Müller, 2013; Schwarz and Müller, 2020). Since the equilibrium constant for the reaction is close to one, the direction of the reaction can be determined by the concentration of the reactants and, thus, formate oxidation to CO_2_ and H_2_ is also possible (Kottenhahn *et al*., 2018). Indeed, both the forward and backward reactions proceed with the highest rates ever found in biological systems (Müller, 2019). Interestingly, the purified HDCR from *A. woodii* can produce formate from CO in the presence of the CODH enzyme of *A. woodii* (Schuchmann and Müller, 2013) and recently we have also shown that whole cells of *T. kivui* convert syngas with high rates into formic acid. Notably, CO was consumed during this process and converted to formate (Schwarz and Müller, 2020). Here, we have built on this existing knowledge to analyse formate production from CO in the presence or absence of Na^+^ and bicarbonate ions. Additional mutagenesis studies should help to give a more detailed understanding of CO‐based formate production in the metabolism of *A. woodii* and *T. kivui*.

## Results

### Whole cell biocatalysis for CO‐dependent acetate and formate production

#### Experiments with *A.woodii* wild type

**Fig. 1:**
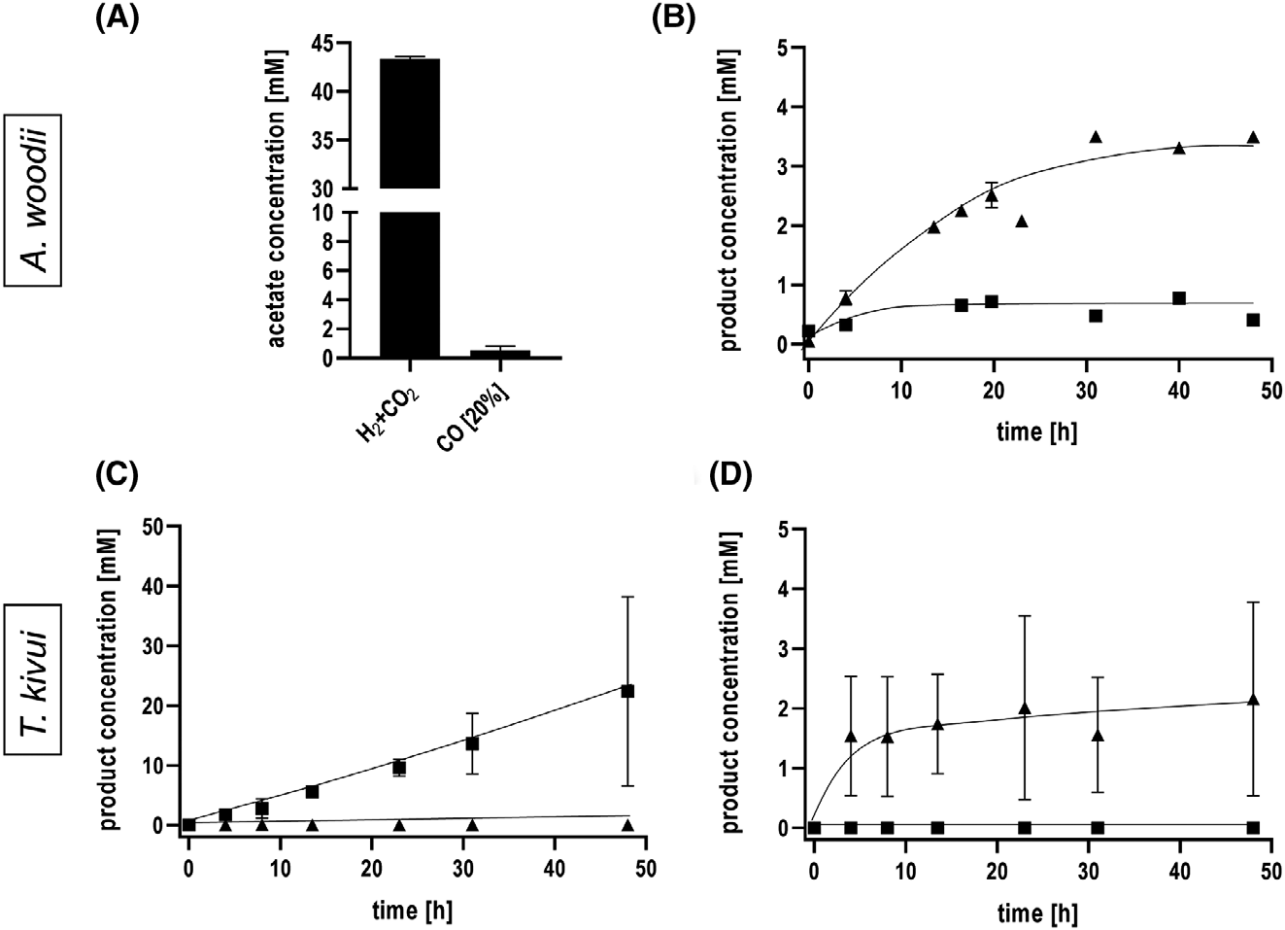
Conversion of CO to formate and acetate by whole cells of *A. woodii* and *T. kivui*. Upper panel (A and B): Cells of *A. woodii* were grown on 20 mM fructose to the late exponential growth phase. The harvested and washed cells were resuspended in buffer A (200 mM imidazole, 20 mM MgSO_4_, 20 mM KCl, 2 mM DTE, 4 µM resazurin, pH 9.0) to a final protein concentration of 2 mg ml^−1^ in anoxic serum bottles. For the H_2_ + CO_2_ experiments, a protein concentration of 1 mg ml^−1^ in buffer B (50 mM imidazole, 20 mM MgSO_4_, 20 mM KCl, 2 mM DTE, 4 µM resazurin, pH 7.0) was used. The cells were incubated with 20% CO (80% N_2_ as makeup gas) or H_2_ + CO_2_ (80:20% [v/v]) at 2 × 10^5^ Pa in a shaking water bath at 30 °C. (A) CO and H_2_ + CO_2_ conversion to acetate in the presence of 20 mM NaCl after 48 h and (B) CO conversion to formate in the absence of NaCl over the time. Lower panel (C and D): Cells of *T. kivui* (pre‐grown on sugar) were grown on 28 mM glucose to the late exponential growth phase. The harvested and washed cells were resuspended in buffer (50 mM imidazole, 20 mM MgSO_4_, 20 mM KCl, 2 mM DTE, 4 µM resazurin, pH 7.0) to a final protein concentration of 1 mg ml^−1^ in anoxic serum bottles. The cells were incubated with 20% CO (80% N_2_ as makeup gas) at 2 × 10^5^ Pa in a shaking water bath at 60 °C. CO conversion to (C) acetate in the absence of KHCO_3_ and (D) formate in the presence of 300 mM KHCO_3_. Triangles up, formate; squares, acetate. Shown are data from two biological replicates. All data points are mean ± SD, *N* = 2.

*Acetobacterium woodii* does not grow on CO alone, but resting cells are known to produce acetate from CO (Kerby *et al*., 1983; Diekert *et al*., 1986; Genthner and Bryant, 1987; Bertsch and Müller, 2015b). Indeed, we observed in this study that cells pre‐grown on fructose produced acetate from CO as electron and carbon source, albeit in small amounts. Under an atmosphere of 20% CO, 0.53 mM acetate was formed after 48 h compared to 42 mM acetate from H_2_ + CO_2_ (Fig. 1A). Chemiosmotic energy conservation in *A. woodii* requires the presence of Na^+^ since the respiratory enzyme, the ferredoxin:NAD^+^ oxidoreductase (Rnf complex), requires Na^+^ for activity and translocates Na^+^ across the membrane thereby establishing a Na^+^ gradient for ATP synthesis (Fritz and Müller, 2007; Biegel and Müller, 2010). In the absence of Na^+^, cells are no longer able to synthesize net ATP and, in general, no acetate is produced. When Na^+^ was omitted in resting cells of *A. woodii* using CO as substrate, little acetate was formed (0.7 mM) but formate (3.4 mM) was now the dominant product; the specific rate of formate production was 0.2 mmol g^−1^ h^−1^ (Fig. 1B).

#### Experiments with *T. kivui* wild type

**Fig. 2:**
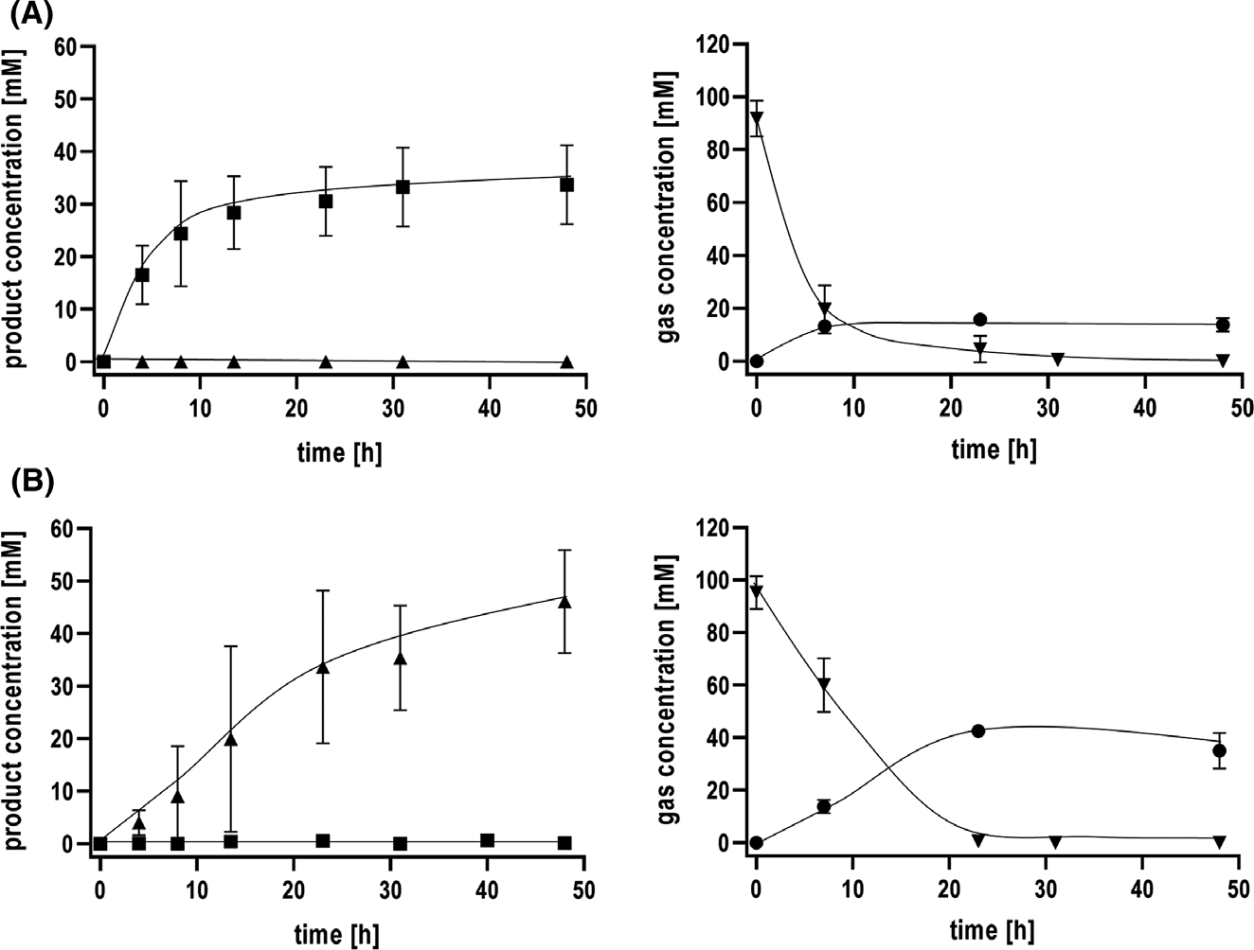
CO‐dependent acetate and formate production by CO‐adapted cells of *T. kivui*. The experiments were performed as described before. Cells of *T. kivui* adapted on 50% CO were supplemented with 20% CO (80% N_2_ as makeup gas) at 2 × 10^5^ Pa as substrate. (A) Production of acetate with the corresponding gas consumption/production and (B) CO conversion to formate in the presence of 300 mM KHCO_3_ with the corresponding gas consumption/production. Triangles up, formate; squares, acetate; triangles down, CO; circles, H_2_. Shown are data from two biological replicates. All data points are mean ± SD, *N* = 2.

Resting cells of *T. kivui* grown on glucose produced acetate from CO with a rate of 0.43 mmol g^−1^ h^−1^ (Fig. 1C), but no formate. However, when the energy metabolism was uncoupled from the WLP by bicarbonate (Schwarz and Müller, 2020), acetate was no longer produced but formate instead (~0.16 mmol g^−1^ h^−1^), up to 2 mM in average (Fig. 1D). By mechanisms that are still not understood, *T. kivui* can be adapted to grow on CO as sole carbon and energy source after several rounds of transfer in media containing increasing amounts of CO (Weghoff and Müller, 2016). When cells were pre‐grown on CO (50%), rates of acetate and formate production from CO were dramatically increased (Fig. 2). The specific acetate production rate in the energetically coupled cells was 3.27 mmol g^−1^ h^−1^, and the specific formate production rate in the energetically uncoupled cells increased by a factor of 8 to 1.34 mmol g^−1^ h^−1^. CO was completely consumed in both cases, and CO consumption was much faster in the energetically coupled, acetate‐forming cells compared to the uncoupled, formate‐producing cells. Under both conditions, hydrogen production was observed, but the uncoupled, formate‐producing cells produced twice as much of molecular hydrogen.

### CO conversion to formate by *T. kivui* at elevated CO concentrations

**Fig. 3:**
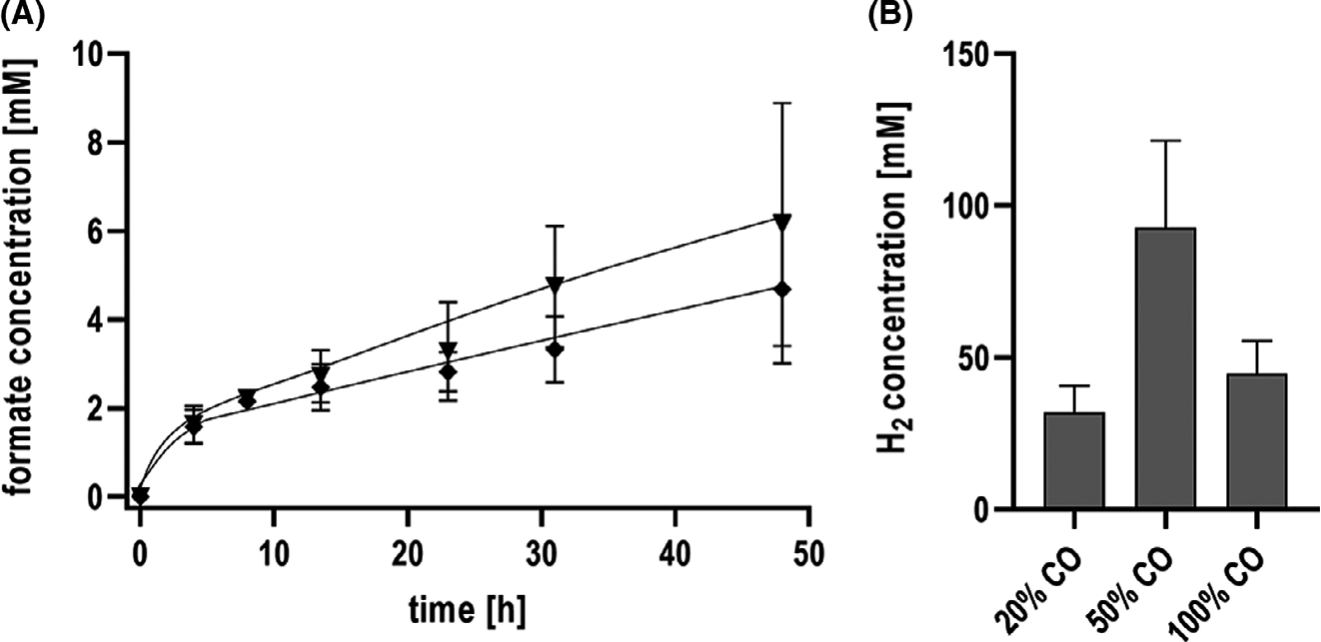
CO conversion to formate under increased CO concentrations. The experiments were performed with cells of *T. kivui* adapted on 50% CO using 300 mM KHCO_3_. (A) Production of formate from 50% (triangles down) and 100% (diamonds) CO. (B) Corresponding H_2_ production on different CO concentrations. Shown are data from two biological replicates. All data points are mean ± SD, *N* = 2.

Next, we tested higher concentrations of 50 and 100% CO as substrate for the production of formate (Fig. 3A). The highest specific formate production rates (1.34 mmol g^−1^ h^−1^) and formate titres (46 mM) were reached using 20% CO. With 50% and 100% CO, formate production rates of only 0.24 and 0.22 mmol g^−1^ h^−1^ and formate titres of only 6.8 mM and 4.7 mM were reached. Clearly, increasing CO concentrations resulted in a reduction of formate production. Only H_2_ production had its peak at 50% CO (Fig. 3B).

### Analysis of CO conversion by mutants

#### Experiments with *T. kivui* mutants

**Fig. 4:**
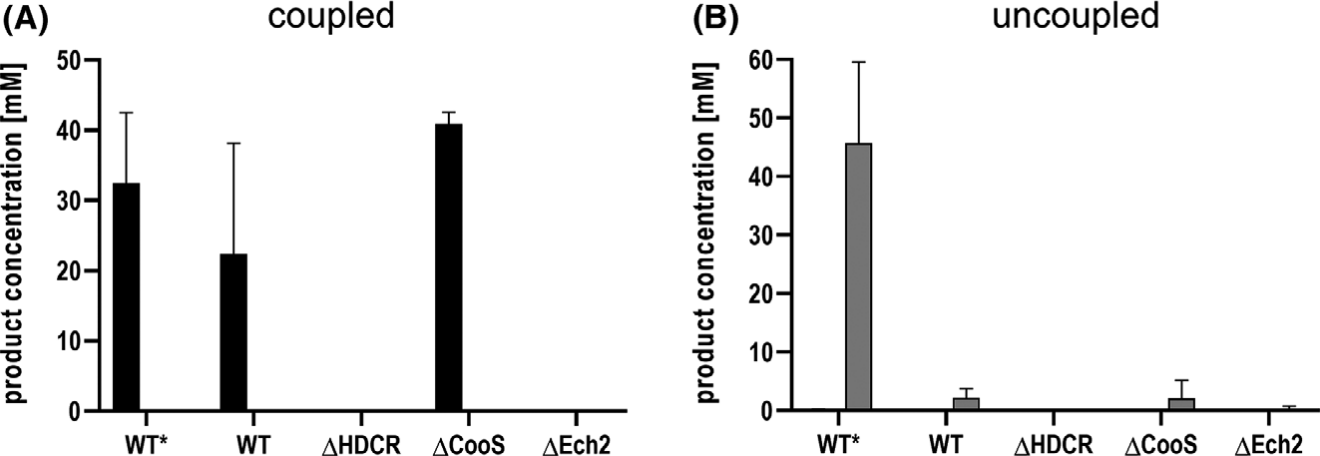
Effect of gene deletions on acetate and formate production by resting cells of *T. kivui*. Cells of *T. kivui* wild type (WT), Δ*hdcr*, Δ*cooS* and Δ*ech2* were grown on 28 mM glucose. Δ*hdrc* had additional 50 mM formate as growth substrate. For the experiments, the cells were incubated with 20% CO (80% N_2_ as makeup gas) at 2 × 10^5^ Pa and the product formation was investigated in (A) coupled or (B) uncoupled cells (additional 300 mM KHCO_3_). WT*, wild‐type strain adapted on 50% CO; WT, non‐CO‐adapted wild‐type strain pre‐grown on glucose; Δ*hdcr*, deletion of the hydrogen‐dependent CO_2_ reductase; Δ*cooS,* deletion of the monofunctional CO dehydrogenase; Δ*ech2*, deletion of the membrane‐bound energy‐conserving hydrogenase. Black bars, acetate; grey bars, formate. Shown are data from two biological replicates. All data points are mean ± SD, *N* = 2.

**Fig. 5:**
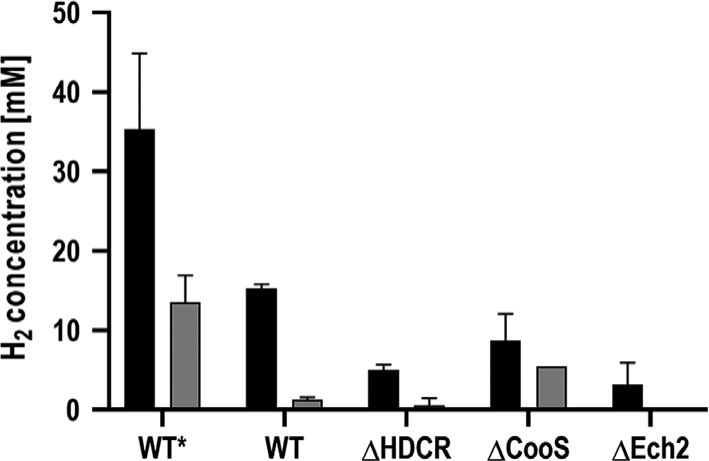
Effect of gene deletions on H_2_ production by resting cells of *T. kivui*. Resting cells of *T. kivui* wild type (WT), CO‐adapted wild type (WT*), Δ*hdcr*, Δ*cooS* and Δ*ech2* were prepared as described before. H_2_ production was monitored during the cell suspension experiments using 20% CO (80% N_2_ as makeup gas) at 2 × 10^5^ Pa as substrate. Black bars, H_2_ production of coupled cells; grey bars, H_2_ production of uncoupled cells. Shown are data from two biological replicates. All data points are mean ± SD, *N* = 2.

To get a deeper look into the enzymes involved in formate production from CO, mutants of *T. kivui* were analysed. The generation and physiological characterization of the ∆*cooS* (TKV_c08080) and ∆*ech2* (TKV_c19680‐TKV_c19750) mutants of *T. kivui* which are lacking the monofunctional CO dehydrogenase (CooS) or energy‐conserving hydrogenase (Ech2) will be described elsewhere; the HDCR deletion mutant (Jain *et al*., 2020) as well as the genetic system (Basen *et al*., 2018) has recently been described in detail. All mutants were generated in the *pyrE*‐deficient uracil‐auxotrophic strain *T. kivui* TKV002, which is a direct daughter strain of *T. kivui* DSM2030, and the generation of all *T. kivui* mutants in this study was based on the same, previously reported genetic system (Basen *et al*., 2018). The ∆*cooS* mutant was generated from a CO‐adapted strain whereas the two other mutants were generated in a glucose‐adapted strain. In this study, all three mutants were grown in complex medium with 28 mM glucose, and the ∆*hdcr* strain with additional 50 mM formate as electron acceptor (Jain *et al*., 2020). Resting cells were then prepared from exponentially grown cultures to analyse their ability for CO (20%) conversion to acetate or formate. As expected, the ∆*hdcr* strain (TKV_c19960‐TKV_c19990) was neither able to produce acetate nor formate from CO, underlining the essentiality of the HDCR complex in the WLP (Fig. 4). The loss of product formation is consistent with a loss of CO consumption (data not shown). Only small amounts of H_2_ (5 mM) were produced (Fig. 5). *T. kivui* has two sets of genes each encoding a membrane‐bound, energy‐conserving hydrogenase (Ech) that catalyses reduction of protons to H_2_ with electrons derived from reduced ferredoxin (Schölmerich and Müller, 2019). The ∆*ech2* strain was no longer able to produce acetate from 20% CO (Fig. 4), and the H_2_ production dramatically decreased by 80% to 3 mM H_2_ (Fig. 5). Furthermore, only traces of formate (0.3 mM) were produced in uncoupled cells and no H_2_ production was observed overall (Figs 4 and 5). These experiments clearly demonstrate a vital function of Ech2 in electron flow from CO to the WLP.

Interestingly, resting cells of the mutant lacking the *cooS* genes were still able to consume CO and to produce acetate. Here, the specific acetate formation rates even increased by a factor of 1.5 to 4.9 mmol g^−1^ h^−1^. 20% CO was completely used up after 23 h, and 8.7 mM H_2_ was produced. When CO‐adapted cells of the wild‐type strain were incubated under uncoupled conditions with CO, formate production was increased dramatically from 2 mM to 45 mM as previously described (Fig. 2B). When the *cooS* gene was deleted, formate production from CO was nearly abolished. Uncoupled cells of the ∆*cooS* mutant produced 2 mM formate like the wild type, and only 5.4 mM H_2_ evolution was observed within 48 h (Figs 4 and 5).

#### Experiments with *A. woodii* mutants

For *A. woodii*, we analysed three mutants in which the HDCR gene cluster (Awo_c08190‐Awo_c08260), the Rnf gene cluster (Awo_c22060‐Awo_c22010) coding for the Na^+^‐dependent membrane‐bound respiratory enzyme and the HydBA gene cluster (Awo_c26980‐Awo_c26970) coding for the soluble electron‐bifurcating hydrogenase were deleted. The latter two mutants and the used genetic system have been described in detail (Westphal *et al*., 2018; Wiechmann *et al*., 2020), and the generation of the HDCR mutant was based on the previously reported genetic system. The generation and characterization of the HDCR mutant will be described elsewhere.

First and as a control, we tested the effect of gene deletions on product formation from H_2_ + CO_2_ (Fig. S1). As expected, the wild type produced high amounts of acetate from H_2_ + CO_2_ only in the presence of Na^+^ and formate production increased in the absence of Na^+^. Since the Rnf complex is directly involved in the bioenergetics of *A. woodii* by generating an electrochemical Na^+^‐ion gradient, the dramatic difference in acetate production as a function of Na^+^ was revoked in the ∆*rnf* mutant. The amount of produced formate stayed the same. The hydrogenase is essential for growth on H_2_ + CO_2_ (Wiechmann *et al*., 2020), and accordingly no acetate was formed; formate production increased slightly.

**Fig. 6:**
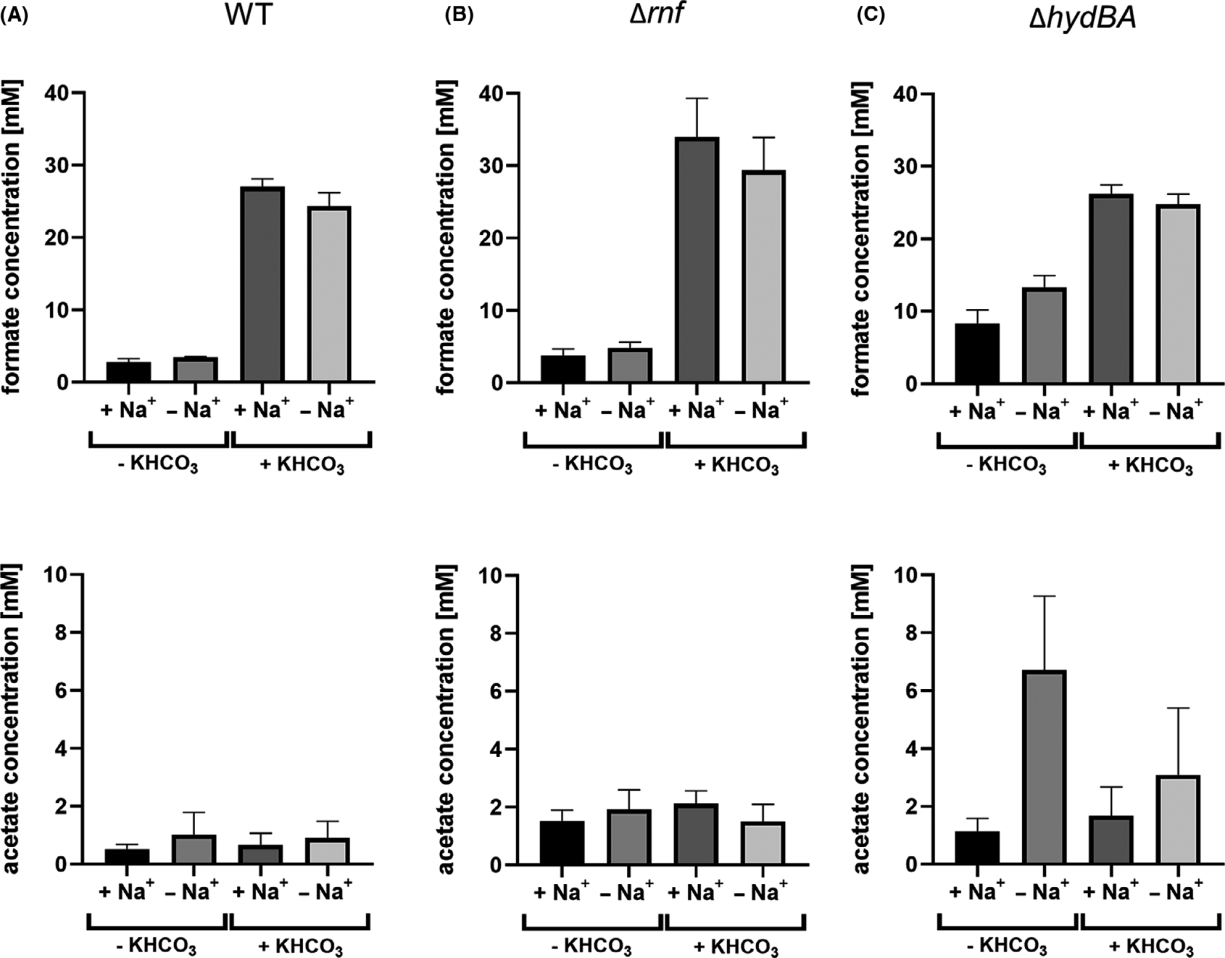
Effect of gene deletions on product formation from 20% CO by whole cells of *A. woodii*. Resting cells of *A. woodii* WT, Δ*rnf* or Δ*hydBA* were prepared as described before. Formate and acetate production was determined after 48 h in the cell suspension experiments with 20% CO (80% N_2_ as makeup gas) at 2 × 10^5^ Pa as substrate using (A) *A. woodii* WT, (B) Δ*rnf* or (C) Δ*hydBA*. +Na^+^, additional 20 mM NaCl, +KHCO_3_, additional 300 mM KHCO_3_; −Na^+^, no NaCl was added; −KHCO_3_, no KHCO_3_ was added. Shown are data from two biological replicates. All data points are mean ± SD, *N* = 2.

The Δ*hdcr* mutant was again no longer able to produce acetate or formate from CO, and CO utilization was not observed (data not shown). The wild type of *A. woodii* produced only little formate from CO in the presence (2.9 mM formate) or absence (3.4 mM formate) of Na^+^ (Fig. 6A). As seen before (Schuchmann and Müller, 2013), the addition of bicarbonate dramatically stimulated formate production by the HDCR in the wild‐type strain but also in the two mutant strains ∆*hydBA* and ∆*rnf* (Fig. 6). The addition of bicarbonate under Na^+^ limiting conditions increased the specific formate production rate in the wild‐type strain by a factor of 4.1. Cell suspension experiments without additional bicarbonate in the reaction buffer will hereinafter be called ‘under CO_2_‐limiting conditions’. Independent of the presence or absence of Na^+^ ions and/ or bicarbonate, wild‐type cells of *A. woodii* produced only traces of acetate (Fig. 6A). The ∆*rnf* mutant showed a slight increase (38%) in formate production compared to the wild‐type strain under CO_2_‐limiting conditions. Amounts of acetate produced were still low but a little higher as in the wild type (Fig. 6B). Interestingly, in the absence of Na^+^ and under CO_2_‐limiting conditions the Δ*hydBA* mutants showed the clearest increase in formate production from CO with rates of 0.48 mmol g^−1^ h^−1^. Here, the final formate titre increased dramatically, reaching 13.3 mM formate after 48 h. This corresponds to an increase in formate by 300% compared to the wild‐type strain (Fig. 6C). In addition to formate, 6.72 mM of acetate was produced as a side product in the mutant strains of Δ*hydBA*, which is roughly 10 times higher compared to the wild type (0.65 mM).

In the wild‐type strain, the rate of formate production increased by 310% to 0.78 mmol g^−1^ h^−1^ and the final titre increased by 610 % to 24.2 mM in the presence of bicarbonate (Fig. S2). This is 82% more compared to the ∆*hydBA* and 390% more to the ∆*rnf* mutants under CO_2_‐limiting conditions. Although formate production in the ∆h*ydBA* was already higher than in the wild type in the absence of bicarbonate, addition of bicarbonate stimulated formate formation even more. In the presence of 300 mM bicarbonate, the ∆*rnf* mutant showed the highest formate production rate of 1.22 mmol g^−1^ h^−1^ and reached the highest final formate titre of 34.5 mM after 48 h. The ∆*hydBA* mutant reached equal dimensions of formate titres (24.8 mM) and a similar range in production rates (0.55 mmol g^−1^ h^−1^) compared to the wild type. The amount of acetate produced in all three strains, wild type, ∆*hydBA* and ∆*rnf*, was analysed to be 0.9, 3.11 and 1.5 mM, respectively. A summary of the specific formate production rates in the absence or presence of bicarbonate and/or Na^+^ using the mutants or wild‐type strain is shown in Table 1.

**Table 1:** Rates of formate production from CO by wild type and mutants of *A. woodii*. An atmosphere of 20% CO was used; makeup gas was N_2_. Shown are data from two biological replicates. All data points are mean (± SD, *N* = 2).

	Specific formate production rates [mmol g^−1^ h^−1^]			
	Without 300 mM KHCO_3_		Additional 300 mM KHCO_3_	
	+Na^+^	Without Na^+^	+Na^+^	Without Na^+^
Wild type	0.17 (± 0.03)	0.19 (± 0.02)	0.68 (± 0.07)	0.78 (± 0.13)
∆*rnf*	0.35 (± 0.18)	0.48 (± 0.27)	1.44 (± 0.14)	1.22 (± 0.16)
∆*hydBA*	0.35 (± 0.02)	0.39 (± 0.05)	0.94 (± 0.17)	0.55 (± 0.07)

+Na^+^, additional 20 mM of NaCl was used in the reaction buffer.

### CO consumption and formate production by *A. woodii* ∆*hydBA*

**Fig. 7:**
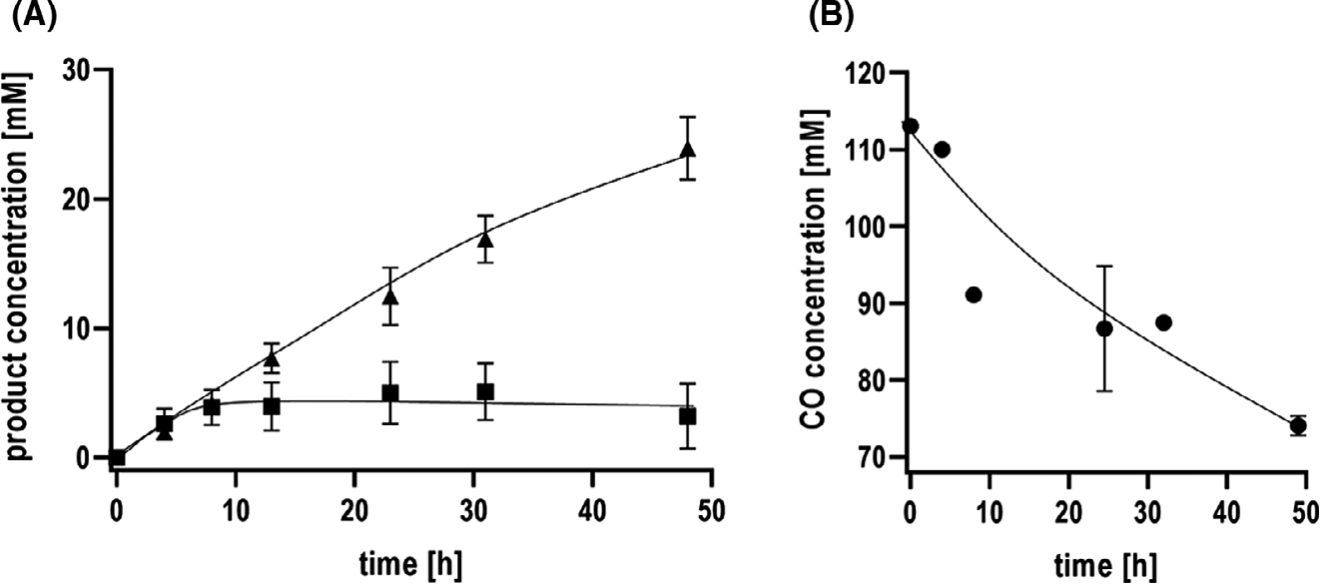
Formate production from CO by *A. woodii ΔhydBA*. Cell suspensions in 200 mM imidazole, 20 mM MgSO_4_, 20 mM KCl, 2mM DTE, 4 µM resazurin, pH 7.0 without an additional source of Na^+^. Cells were incubated with 20% CO (80% N_2_ as makeup gas) at 2 × 10^5^ Pa and with additional 300 mM KHCO_3_. (A) Formate (triangles up) and acetate (squares) production and corresponding (B) CO (circles) consumption. Shown are data from two biological replicates. All data points are mean ± SD, *N* = 2.

CO utilization and formate formation were further analysed in detail in the presence of bicarbonate and under Na^+^ limiting conditions using the Δ*hydBA* mutant. The cell suspension converted 20% CO to 24.8 mM formate with a formate production rate of 0.55 mmol g^−1^ h^−1^. In addition, 3.81 mM of acetate was produced (Fig. 7A). Simultaneously, 39 mM of CO was consumed (Fig. 7B). CO concentrations up to 100% were tolerated without significant loss of formate production activities (Fig. S3).

## Discussion

### CO utilization pathway in *T. kivui*

The two acetogenic bacteria *A. woodii* and *T. kivui* were shown in this study to work as whole cell biocatalysts for the conversion of the toxic gas CO either to acetate or to formate, the latter with rates which are so far the highest in the literature (Rother and Metcalf, 2004; Henstra *et al*., 2007a; Mayer *et al*., 2018; Hwang *et al*., 2020). This allows the production of two interesting bulk chemicals from the highly abundant and toxic industrial gas CO as initial substrate. Noteworthy, production of formate goes along with little side products, that is high selectivity. In contrast to other CO utilizers such as *C. autoethanogenum*, product formation is thus more controllable. Furthermore, the mechanism responsible for the CO‐based formate production was investigated in a closer look at using mutants with defects in key intracellular enzyme activities.

The key enzyme in anaerobic as well as aerobic microbial CO utilization is the carbon monoxide dehydrogenase (CODH). This enzyme catalyses the oxidation of CO to CO_2_ and protons/electrons (Eq. 3).

(3) $$1\text{CO}+1H_{2}O\Leftrightarrow 1{\text{CO}}_{2}+2H^{+}+2e^{-}$$

**Fig. 8:**
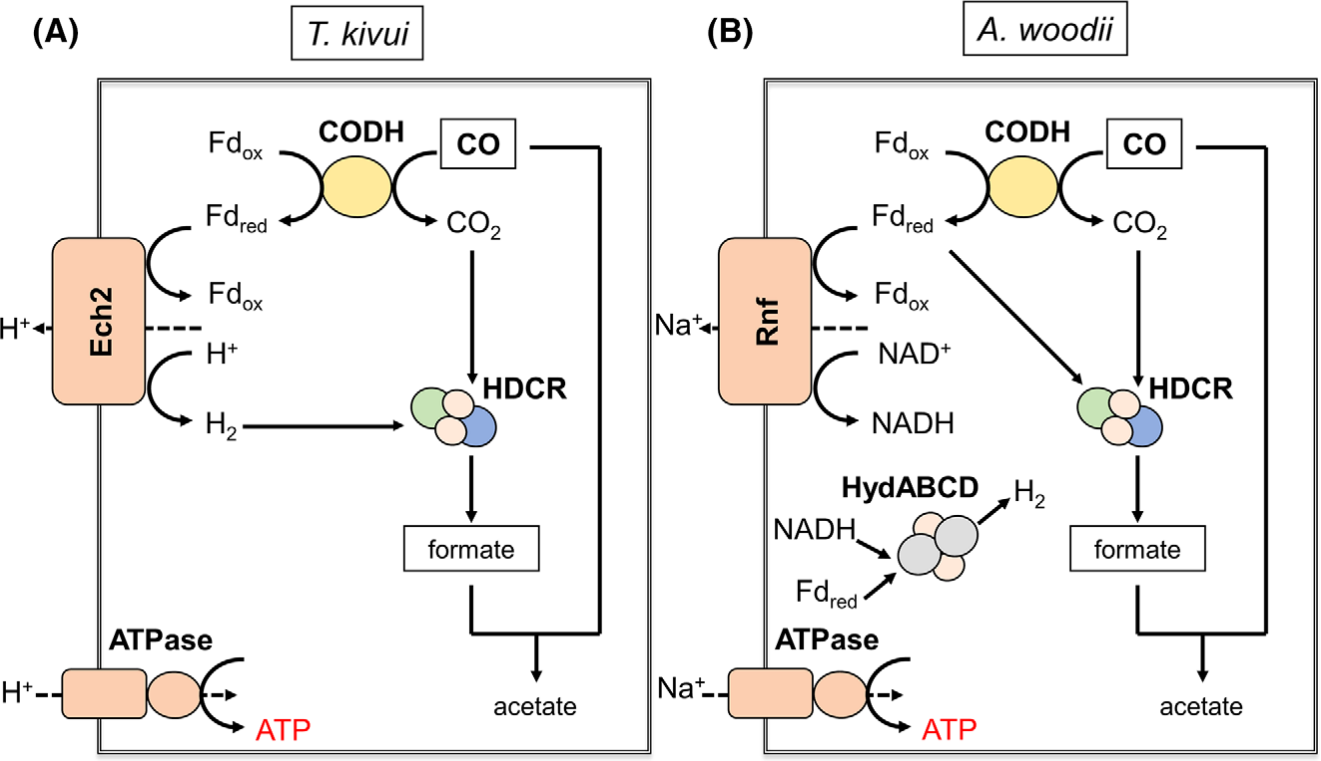
Schematic model of the CO utilization pathway in the two acetogenic bacteria *T. kivui* and *A. woodii*. CODH, CO dehydrogenase; HDCR, Hydrogen‐dependent CO_2_ reductase; Ech, Energy‐conserving hydrogenase complex; Rnf, Rhodobacter nitrogen fixation complex; HydABCD, Electron‐bifurcating hydrogenase; Fd_red_, reduced ferredoxin; Fd_ox_, oxidized ferredoxin.

The electron acceptors are diverse and in the two acetogenic bacteria *A. woodii* and *Moorella thermoacetica* the CODH was purified and shown to use ferredoxin as electron acceptor (Ragsdale *et al*., 1983). The same can be assumed for *T. kivui*. However, there are two CO dehydrogenases present in *T. kivui*, the monofunctional CODH (CooS) and the bifunctional CODH/ACS (Hess *et al*., 2014). Deletion of *cooS* did not reduce but stimulated acetate formation compared to the wild type, leading to the conclusion that the CODH/ACS alone is able to oxidize CO and to catalyse acetate formation from CO. The dispensability of the monofunctional CO dehydrogenases in autotrophy was also shown in mutagenesis studies for *C. autoethanogenum* (Liew *et al*., 2016a). In the uncoupled system of *T. kivui* that does not allow for acetate synthesis, the wild type produced only little formate. However, the CO‐adapted strain produced much more formate and deletion of *cooS* almost abolished formate production. This is consistent with the hypothesis that CooS is essential for CO‐coupled formate production in CO‐adapted cells. The ferredoxin reduced by CooS is then oxidized by Ech2, as evident from the complete loss of formate and acetate production and dramatic reduction in production of molecular hydrogen in the ∆*ech2* mutant. The HDCR uses H_2_ as preferred reductant, but can also use reduced ferredoxin as reductant, albeit with ~ 95% less activity. Since a ∆*ech2* mutant does no longer produce formate or acetate, it has to be concluded that the HDCR *in vivo* requires H_2_ that cannot be replaced by reduced ferredoxin. The same has been observed very recently for the HDCR from *A. woodii in vivo* (Wiechmann *et al*., 2020). Last, a ∆*hdcr* mutant does not produce formate. In sum, these data are consistent with the following pathway of formate production from CO: CO is oxidized to CO_2_ by CODH/ACS or CooS; the former dominates in non‐CO‐adapted cells, the latter in CO‐adapted cells. CO oxidation is coupled to reduction of ferredoxin which is oxidized by Ech2 to produce molecular hydrogen. CO_2_ is then reduced by the HDCR with electrons derived from H_2_ to formate (Fig. 8A).

### CO utilization pathway in *A. woodii*

In *A. woodii* the situation is not as clear. CO is oxidized either by CooS (Awo_c19050) or CODH/ACS thereby reducing ferredoxin. Reduced ferredoxin is then reoxidized by the Rnf complex to reduce NAD^+^. NADH and Fd^2−^ are then used by the electron‐bifurcating hydrogenase to reduce protons to molecular hydrogen, which can drive CO_2_ reduction to formate via the HDCR. But since resting cells of the ∆*rnf* mutant as well as the wild‐type strain under Na^+^ limiting conditions are still able to produce acetate from H_2_ + CO_2_ (Fig. S1), we have to assume that residual ATP pools enable the cells to produce small amounts of acetate (Westphal *et al*., 2018) without the need for an electrochemical Na^+^ gradient. Furthermore, the HDCR seems to be able to use reduced ferredoxin as an alternative electron donor for the reduction of CO_2_ to formate, especially in resting cells of *A. woodii*. As previously mentioned, the use of reduced ferredoxin as reductant was already shown for the purified HDCR in *in vitro* experiments (Schuchmann and Müller, 2013). This is not in contradiction to the postulated hydrogen cycling mechanism in *A. woodii* where hydrogen is directly used as reductant for CO_2_ reduction in growing cells of *A. woodii* (Wiechmann *et al*., 2020). The use of reduced ferredoxin (Fd^2‐^) from the HDCR could also explain the increasing amounts of formate produced in the ∆*hydBA* and ∆*rnf* mutant, since in each mutant only one Fd^2‐^ consuming module is active, thus increasing the electron‐pressure in form of reduced ferredoxin towards the HDCR, resulting in increased formate titres. In the case of ∆*hydBA*, a putative electron‐loss by the electron‐bifurcating, hydrogen‐producing hydrogenase is not possible and could, therefore, result in even higher formate titres. Hydrogen evolution could not be observed in any strain tested. This is not surprising since until now, hydrogen evolution and hydrogen cycling could only be observed for cultures of *A. woodii* which were cultivated in a stirred‐tank bioreactor (Wiechmann *et al*., 2020). Unfortunately, the production of 6.72 mM acetate in the ∆*hydBA* mutant strain under CO_2_‐ and Na^+^‐limiting conditions cannot be resolved in this study and remains uncertain. Based on the metabolic and enzymatic knowledge to date, an additional enzyme for the conversion of reduced ferredoxin to NADH seems to be necessary to explain the production of acetate from CO under the given conditions. Nevertheless, in sum the data allow to postulate the following pathway of formate production from CO in resting cells of *A. woodii*: CO is oxidized to CO_2_ by CODH/ACS or CooS, generating reduced ferredoxin. Ferredoxin can then either be used by the Rnf complex, the electron‐bifurcating hydrogenase or the HDCR complex, the latter one involved in ferredoxin‐based CO_2_ reduction to formate (Fig. 8B). Depending of the electron‐pressure (as Fd^2‐^), the HDCR catalysed formate production, especially the specific formate formation rates as well as formate titres, could differ.

### Bicarbonate stimulates formate production in *A. woodii*

All three strains tested, wild type, ∆*hydBA* and ∆*rnf* of *A. woodii*, have in common that additional bicarbonate dramatically stimulated formate formation. The addition of bicarbonate leads to a fast interconversion of bicarbonate and CO_2_ by the carbonic anhydrase of resting cells (Braus‐Stromeyer *et al*., 1997), thus increasing the available amount of substrate (CO_2_) for the HDCR reaction. Since the equilibrium constant of the hydrogen‐dependent CO_2_ reduction is close to one, the state of the chemical equilibrium can be easily affected. As seen for *T. kivui* (Schwarz and Müller, 2020), bicarbonate could also potentially influence enzymes in the WLP or enzymes involved in energy conservation/ATP generation that inhibit the further conversion of formate to acetate and thereby stimulating the HDCR catalysed Fd^2‐^‐dependent CO_2_ reduction to formate through higher substrate availability of CO_2_.

At the end, we can sum up that mutagenesis studies in *A. woodii* and *T. kivui* revealed a difference in the electron donor (Fd^2‐^ or H_2_) as well as in the electron flow for CO‐based formate production in resting cells of these organisms. Not only the mutations but also the presence/absence of Na^+^ and bicarbonate ions affected the specific formate production rates as well as final formate/acetate titres.

## Experimental procedures

### Organism and cultivation

*Thermoanaerobacter kivui* LKT‐1 (DSM 2030) and its mutants ∆*hdcr*, ∆*ech2* and ∆*cooS* were cultivated at 66 °C under anoxic conditions in complex medium (Weghoff and Müller, 2016) using 1‐l flasks (Müller‐Krempel, Bülach, Switzerland). The flasks contained 500 ml media for heterotrophic cultivation and 200 ml media for autotrophic cultivation to increase the gas‐to‐liquid ratio. Media were prepared under anoxic conditions as described before (Hungate, 1969; Bryant, 1972). Glucose (28 mM) or CO (50% CO, 40% N_2_ and 10% CO_2_ [v/v] at 2 × 10^5^ Pa) were used as substrate. For the cultivation of *T. kivui* ∆*hdcr,* additional 50 mM formate was used. *Acetobacterium woodii* (DSM 1030) and its mutants ∆*hydBA*, ∆*hdcr* and ∆*rnf* were cultivated at 30 °C under anoxic conditions in carbonate‐buffered medium (Heise *et al*., 1989). The medium was prepared as described before (Hungate, 1969; Bryant, 1972). Fructose (20 mM) was used as growth substrate for all cultivations, and additional 50 mg l^−1^ uracil was added to the *pyrE* deletion mutants. The growth media of *A. woodii* ∆*hdcr* and ∆*hydBA* were supplemented with additional 40 mM formate. Growth was followed by measuring the optical density at 600 nm with an UV/Vis spectrophotometer.

### Preparation of resting cells and cell suspension experiments

Preparation of resting cells was performed under strictly anoxic conditions in an anaerobic chamber (Coy Laboratory Products, Grass Lake, MI, USA) as described (Heise *et al*., 1992). Cells of *A. woodii* and *T. kivui* were cultivated in 1‐l flasks (Müller‐Krempel, Bülach, Switzerland) to the late exponential growth phase and were harvested by centrifugation at 11500 *g* at 4 °C for 10 min. Afterwards, the cells were washed twice in imidazole buffer (50 mM imidazole, 20 mM MgSO_4_, 20 mM KCl, 4 µM resazurin, 2 mM DTE, pH 7.0). If not otherwise stated, *T. kivui* cells were resuspended in the same imidazole buffer to a final protein concentration of 1 mg/ml. Cells of *A. woodii* were resuspended in 200 mM imidazole buffer (200 mM imidazole, 20 mM MgSO_4_, 20 mM KCl, 4 µM resazurin, 2 mM DTE, pH 9.0) to a final protein concentration of 2 mg ml^−1^. The cell suspensions were transferred to gas‐tight Hungate tubes and were directly used for the subsequent cell suspension experiments. The protein concentration of the cell suspension was determined according to (Schmidt *et al*., 1963).

For determining the conversion of CO in cell suspension experiments of *A. woodii* and *T. kivui*, 60 ml serum bottles (Glasgerätebau Ochs GmbH, Bovenden‐Lenglern, Germany) with N_2_ atmosphere were filled with imidazole buffer (50 mM imidazole, 20 mM MgSO_4_, 20 mM KCl, 4 µM resazurin, 2 mM DTE, pH 7.0 or 200 mM imidazole, 20 mM MgSO_4_, 20 mM KCl, 4 µM resazurin, 2 mM DTE, pH 9.0) and the head space was changed to 20% CO (80% N_2_ as makeup gas), 50% CO (50% N_2_ as makeup gas) and 100% CO with 1 bar overpressure in total. The serum flasks contained a final liquid volume of 5 ml. The serum flasks were pre‐warmed for at least 10 min at 30 °C or at 60 °C for cells of *A. woodii* and *T. kivui*, respectively. If necessary, bicarbonate was added prior to the reaction start. The reaction was started by adding the cell suspension and samples were taken at defined time points.

For acetogenesis from H_2_ + CO_2_ by *A. woodii*, cells were cultivated and harvested as described above. Cell suspensions in imidazole buffer (50 mM imidazole, 20 mM MgSO_4_, 20 mM KCl, 4 µM resazurin, 2 mM DTE, pH 7.0) containing additional 20 mM NaCl or no additional NaCl were incubated in 120 ml serum bottles (Glasgerätebau Ochs GmbH, Bovenden‐Lenglern, Germany) filled with a final volume of 10 ml. A cell concentration corresponding to 1 mg total cell protein per ml and a gas atmosphere of H_2_ + CO_2_ (80:20%, [v/v]) at 1 bar overpressure were used.

### Analytical methods

The concentrations of acetate and formate were measured by high‐performance liquid chromatography (1260 Infinity II LC System) equipped with 1260 Infinity II Quaternary Pump, 1260 Infinity II Vialsampler, 1260 Infinity II Multicolumn Thermostat, 1260 Infinity II Diode Array Detector and 1260 Infinity II Refractive Index Detector (Agilent Technologies, Santa Clara, CA, USA). For sample preparation, cells were spun down by centrifugation at 18 000 *g* for 10 min and the supernatant was filtered via syringe filters (4 mm Millex‐LH Syringe Filters; Merck KGaA, Darmstadt, Germany) into a 400 µl flat bottom glass insert (Agilent Technologies, Santa Clara, CA, USA) of the HPLC vial. A Hi‐Plex H 300 × 7.7 mm column with its precolumn Hi‐Plex H Guard 50 × 7.7 mm (Agilent Technologies, Santa Clara, CA, USA) was used for separation. Filtered and degassed sulphuric acid (5 mM) was used as eluent at a flow rate of 0.6 ml min^−1^. The vial sampler and the oven were kept at 5 °C and 55 °C, respectively. The sample (5 µl) was injected by the auto‐sampler and analysed with a refractive index detector at 55 °C and a diode array detector operating in the range of 200 to 220 nm. The reference cell of the refractive index detector was purged with the eluent prior to analysis. The run time of the sample analysis was 30 min. CO and H_2_ were determined as described before (Weghoff and Müller, 2016; Schwarz *et al*., 2018).

## Conflict of interest

The authors declare that they have no competing interests.

## Author contributions

V.M. designed and supervised the research, analysed the data and wrote the manuscript. F.M.S. designed and supervised the research, performed the experiments, analysed the data and wrote the manuscript. M.B. supervised the research of C.B. S.C. performed the experiments and analysed the data. S.J., A.W. and C.B. generated the mutant strains.

## Supporting information


**Fig. S1.** Effect of gene deletions on product formation from H_2_ + CO_2_ by whole‐cells of *Acetobacterium woodii* in the presence or absence of Na^+^.
**Fig. S2.** Stimulation of formate production by increasing concentrations of bicarbonate.
**Fig. S3.** Influence of various CO concentrations on formate production using resting cells of *A. woodii* ∆hydBA.
