# Supplementary material for: Revealing formate production from carbon monoxide in wild type and mutants of Rnf‐ and Ech‐containing acetogens, Acetobacterium woodii and Thermoanaerobacter kivui
Source: Microb Biotechnol. 2020 Sep 21;13(6):2044–56. doi: 10.1111/1751-7915.13663 (PMC7533326; doi:10.1111/1751-7915.13663)
Supplement: Supplementary file 1 — Fig. S1. Effect of gene deletions on product formation from H2 + CO2 by whole‐cells of Acetobacterium woodii in the presence or absence of Na+. Fig. S2. Stimulation of formate production by increasing concentrations of bicarbonate. Fig. S3. Influence of various CO concentrations on formate production using resting cells of A. woodii ∆hydBA. [file MBT2-13-2044-s001.docx]

**Supporting information**

**Revealing formate production from carbon monoxide in wild type and mutants of Rnf- and Ech-containing acetogens,
*Acetobacterium woodii* and *Thermoanaerobacter kivui***

Fabian M. Schwarz^1^, Sarah Ciurus^1^, Surbhi Jain^1^, Christoph Baum^2^, Anja Wiechmann^1^, Mirko Basen^2^, Volker Müller^1^*

^1^Molecular Microbiology & Bioenergetics, Institute of Molecular Biosciences,

Johann Wolfgang Goethe University, Frankfurt am Main, Germany

^2^Microbiology, Institute of Biological Sciences, University Rostock, Rostock, Germany

^*^Mailing address: Max-von-Laue-Str. 9, 60438 Frankfurt, Germany. Phone +49-6979829507.

Fax +49-69-79829306. E-mail: vmueller@bio.uni-frankfurt.de

**Additional file S1**


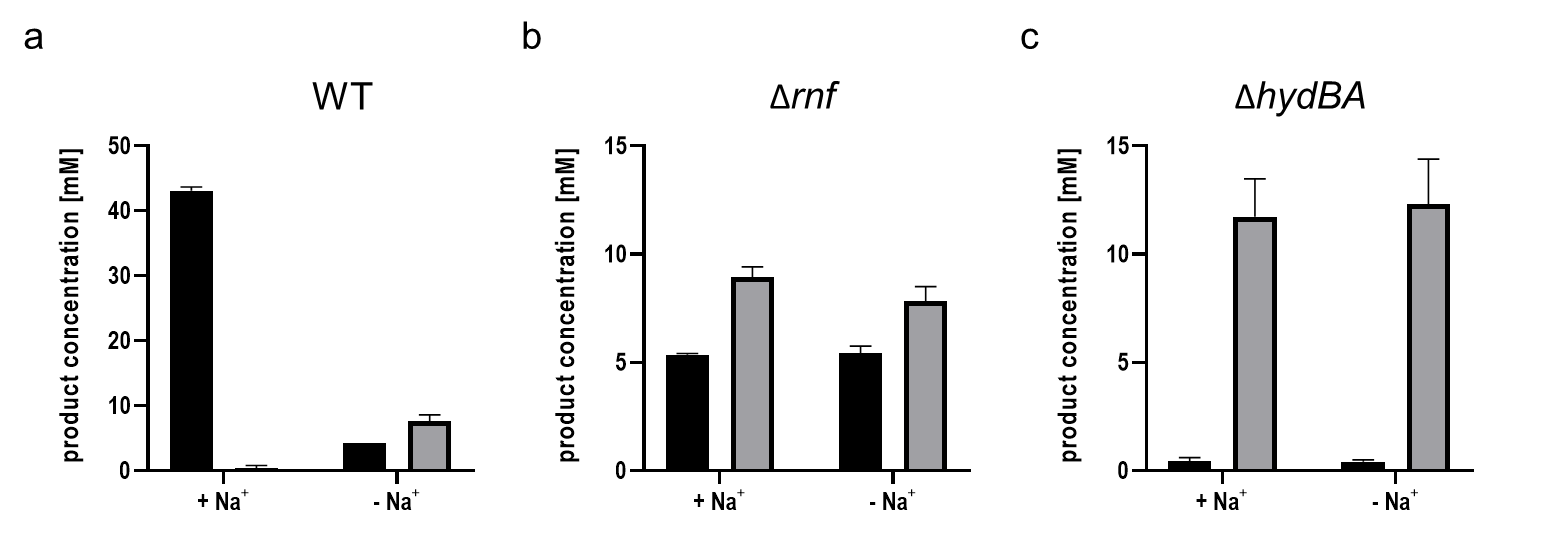


**Figure S1. Effect of gene deletions on product formation from H_2_ + CO_2_ by whole-cells of A. woodii in the presence or absence of Na^+^**. Resting cells of A. woodii WT, ∆rnf or ∆hydBA were prepared as described before in buffer B (50 mM imidazole, 20 mM MgSO_4_, 20 mM KCl, 2mM DTE, 4 µM resazurin, pH 7.0). Formate (grey bars) and acetate (black bars) production was determined after 48 h in cell suspension experiments with H_2_ + CO_2_ (80:20% [v/v]) at 2 x 10^5^ Pa as substrate using **(a)** A. woodii WT, **(b)** ∆rnf or **(c)** ∆hydBA. +Na^+^, additional 20 mM NaCl in the reaction buffer; -Na^+^, no additional NaCl. Shown are data from two biological replicates. All data points are mean ± SD, N=2.

**Additional file S2**


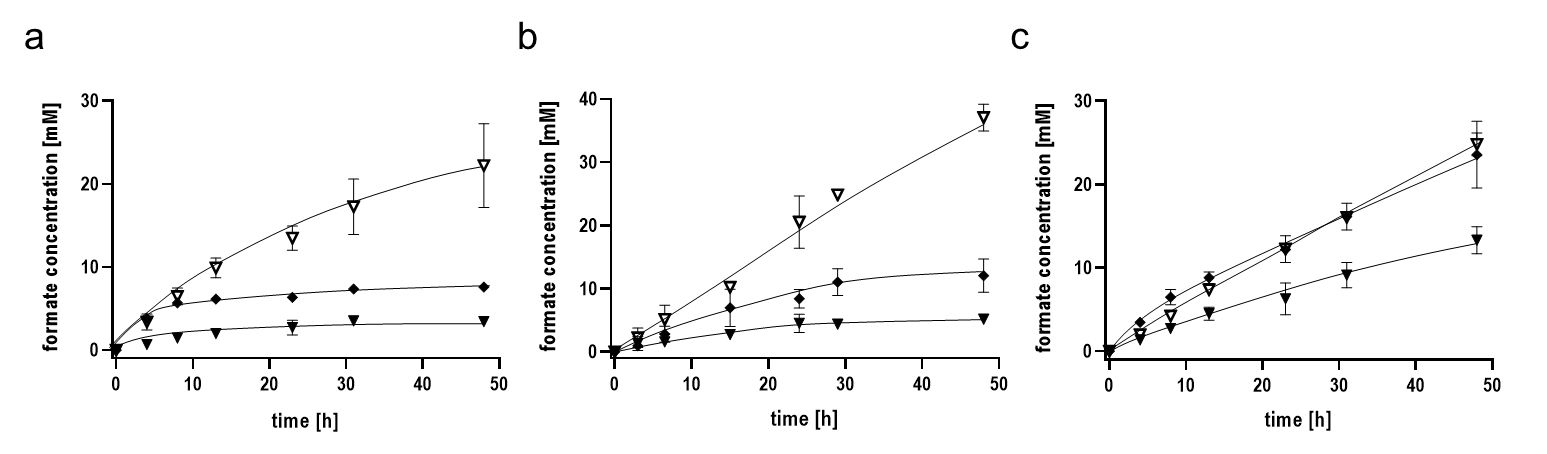


**Figure S2. Stimulation of formate production by increasing concentrations of bicarbonate.** Resting cells of **(a)** A. woodii wildtype, **(b)** Δrnf and **(c)** ΔhydBA were incubated with additional 0 mM (triangles down), 50 mM (diamonds) and 300 mM (open triangles down) KHCO_3_ in the buffer. The experiment was performed in the absence of Na^+^ and by using 20% CO (80% N_2_ as makeup gas) at 2 x 10^5^ Pa as substrate. Shown are data from two biological replicates. All data points are mean ± SD, N=2.

**Additional file S3**


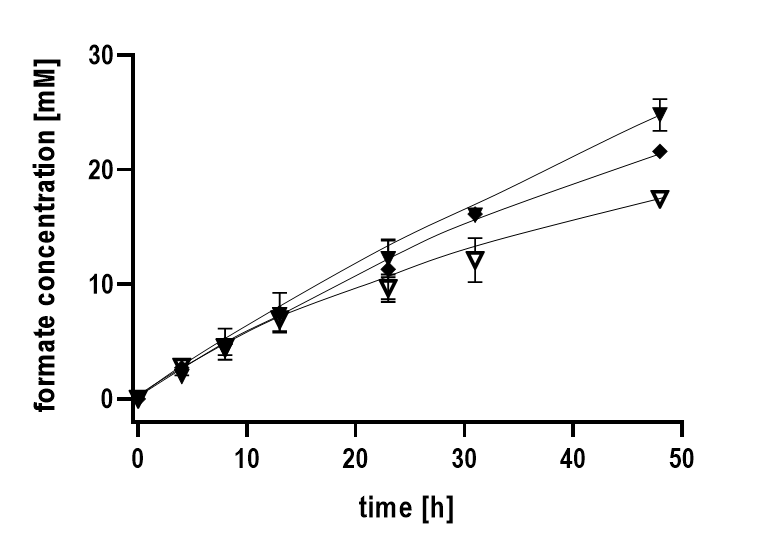


**Figure S3. Influence of various CO concentrations on formate production using resting cells of A. woodii ∆hydBA.** Cells were resuspended in buffer (200 mM imidazole, 20 mM MgSO_4_, 20 mM KCl, 2mM DTE, 4 µM resazurin, pH 7.0) and were incubated with 20% (triangles down), 50% (diamonds) and 100% (open triangles down) CO as substrate. N_2_ was used as makeup gas. The experiments were performed with additional 300 mM KHCO_3_ and in the absence of Na^+^. Shown are data from two biological replicates. All data points are mean ± SD, N=2.
